# Left Atrial Stiffness: A Predictor of Atrial Fibrillation Recurrence after Radiofrequency Catheter Ablation - A Systematic Review and Meta-Analysis

**DOI:** 10.5935/abc.20190040

**Published:** 2019-05

**Authors:** Eduardo Thadeu de Oliveira Correia, Letícia Mara dos Santos Barbetta, Othon Moura Pereira da Silva, Evandro Tinoco Mesquita

**Affiliations:** Universidade Federal Fluminense (UFF), Niterói, RJ - Brazil

**Keywords:** Atrial Fibrillation, Catheter Ablation/methods, Heart Atria, Tachycardia, Paroxysmal, Metanalysis

## Abstract

**Background:**

Radiofrequency catheter ablation (RFCA) is a standard procedure for patients with atrial fibrillation (AF) not responsive to previous treatments, that has been increasingly considered as a first-line therapy. In this context, perioperative screening for risk factors has become important. A previous study showed that a high left atrial (LA) pressure is associated with AF recurrence after ablation, which may be secondary to a stiff left atrium.

**Objective:**

To investigate, through a systematic review and meta-analysis, if LA stiffness could be a predictor of AF recurrence after RFCA, and to discuss its clinical use.

**Methods:**

The meta-analysis followed the MOOSE recommendations. The search was performed in MEDLINE and Cochrane Central Register of Controlled Trials databases, until March 2018. Two authors performed screening, data extraction and quality assessment of the studies.

**Results:**

All studies were graded with good quality. A funnel plot was constructed, which did not show any publication bias. Four prospective observational studies were included in the systematic review and 3 of them in the meta-analysis. Statistical significance was defined at p value < 0.05. LA stiffness was a strong independent predictor of AF recurrence after RFCA (HR = 3.55, 95% CI 1.75-4.73, p = 0.0002).

**Conclusion:**

A non-invasive assessment of LA stiffness prior to ablation can be used as a potential screening factor to select or to closely follow patients with higher risks of AF recurrence and development of the stiff LA syndrome.

## Introduction

Radiofrequency catheter ablation (RFCA) is a standard procedure for the treatment of atrial fibrillation (AF) in patients not responsive to previous treatments.^1^ However, growing evidence has shown lower rates of AF recurrence and AF burden in patients with paroxysmal AF that were submitted to ablation as a first-line therapy option.^2^ In addition to that, progression from paroxysmal AF to persistent AF appears to be delayed by early catheter ablation of AF.^2^ Therefore, catheter ablation has been increasingly considered as a first-line therapy option, which makes it more important to use screening factors to closely follow patients with higher risk of AF recurrence and post-procedural complications.

Recently, the importance of studying left atrial (LA) stiffness has been growing exponentially, since it has been linked to the stiff left atrial syndrome (SLAS), a severe consequence of RFCA.^3^ Moreover, a previous study^4^ showed that an increase in LA pressure is associated with AF recurrence after ablation. Since a high LA pressure may be secondary to an increase in LA stiffness,^5^ LA stiffness itself could be a predictor of AF recurrence after RFCA and, thereby promote a closer follow-up of patients at higher risk of AF recurrence and development of the SLAS. However, no systematic review or meta-analysis has been published to investigate this relationship, although these studies could provide the strongest and the highest quality of evidence.

Therefore, this systematic review and meta-analysis aims to investigate if LA stiffness itself could be a predictor of AF recurrence after RFCA and discusses the clinical usefulness of this new predictor.^6^

## Methods

A systematic review was performed using the criteria established by the Meta-analysis of Observational studies in the Epidemiology Group (MOOSE).

### Search strategy

Two investigators (ETOC, ETM) searched the MEDLINE and the Cochrane Central Register of Controlled Trials databases, until March 2018. We searched for a combination of English terms and Medical Subject Headings (MeSH) descriptors, consisting of seven keywords [("left atrial" OR "left atrium") AND ("stiff" OR "stiffness" OR "compliance") AND ("ablation" OR "pulmonary vein isolation")]. A manual search of references was also used to identify possible studies for inclusion. If necessary, an English translation of the retrieved articles would be obtained. Each title and abstract were independently analysed by the two investigators, who selected the articles which would be relevant to the review. After that, the full texts of the remaining articles were reviewed to select which would be included in the qualitative or quantitative analysis. In case of disagreement, the decision was made by discussion and consensus of the authors.

### Inclusion criteria

We included observational studies (with prospective or retrospective nature) in humans, whose objective was to study the association between LA stiffness and recurrence of AF after the first RFCA.

For qualitative analysis, studies with the following characteristics were included: 1) The study evaluated AF recurrence after the first RFCA in human subjects; 2) Retrospective or prospective observational studies; 3) The mean follow-up period was longer than 6 months; 4) The study included more than 20 subjects.

For the quantitative analysis, we included studies that fulfilled all the previous criteria and reported hazard ratio (HR) and 95% confidence intervals (CI) of LA stiffness as predictors of AF recurrence.

### Quality assessment

The risk of bias in the studies was evaluated using the National Heart, Lung and Blood Institute Quality Assessment Tool for Case Series Studies.^7^ The evaluation was done independently by two raters (ETOC, LMSB), and in case of disagreement the decision was made by consensus of the raters. The following characteristics were assessed: 1) Was the study question or objective clearly stated?; 2) Was the study population clearly and fully described, including a case definition?; 3) Were the cases consecutive?; 4) Were the subjects comparable?; 5) Was the intervention clearly described?; 6) Were the outcome measures clearly defined, valid, reliable, and implemented consistently across all study participants?; 7) Was the length of follow-up adequate?; 8) Were the statistical methods well-described?; 9) Were the results well-described? 

After these characteristics were assessed, the authors gave the studies one of the quality ratings (good, fair or poor). Studies were rated as 'poor' if they met less than three criteria, 'fair' if they met three to five criteria, and 'good' if they met more than five criteria. All four articles selected met almost all the criteria and received a good quality rating by the two raters. The quality assessment of the four studies is reported in Table 1.

**Table 1:** Characteristics of the included studies

Study, year	Region	Study design	Number of Patients	Ablation strategy	Measurement of LA stiffness	Method of AF detection	Follow-up, months	Blanking period, months	Findings	Quality
MachinoOhtsuka et al., 2011	Asia	Prospective case series, single centre	155	PVI	Ratio of the difference between the LA peak v-wave pressure and the LA x-wave pressure nadir of the global S-LAs [(LAP-v - LAP-x) / global S-LAs]	12-lead ECG, arrhythmia-related symptom, 24-hour Holter monitoring and portable ECG monitoring	Mean follow-up period of 33.8 ± 12.2 months (range, 14 to 54 months)	3	LA stiffness index was an independent predictor of recurrence of AF (HR: 2.88; 95% CI: 1.75 to 4.73, p < 0.001)	Good
Park et al., 2015*	Asia	Prospective case series, single centre	334	PVI	Direct measurement of LA pulse pressure (the difference between LAP peak and LAP nadir) and assumed a minimal change in LA volume based on the previous physiologic studies	ECG and 24- or 48-hour Holter monitoring	Mean follow-up period of 16.7 ± 11.8 months (range, 3 to 47 months)	NR	Low LA compliance was independently associated with two fold-higher risk of clinical AF recurrence (HR: 2.202; 95%CI: 1.077 to 4.503; p = 0.031)	Good
Kawasaki et al, 2016	Japan	Prospective, case series, single centre	109	PVI	LA stiffness was obtained by using ePCWP as ePCWP/LA strain obtained by STE	ECG and Holter recordings	At least 12 months	1	LA stiffness index was not a predictor of recurrence of AF (OR: 0.37, 95%, CI: 0.041 to 3.462, p = 0.39)	Good
Khurram et al., 2016†	North America	Prospective, case series, single centre	160	PVI	Ratio of change in LAP to the change in LA volume during passive filling of LA.	24-hour Holter monitoring or 30-day event monitoring	Mean follow-up period of 10.4 ± 7.6 months	3	LA stiffness index was an independent predictor of AF ablation outcome (HR: 8.22; 95% CI: 3.54 to 19.11; p < 0.001)	Good

LA: Left atrial; LAP: Left atrial pressure; AF: atrial fibrillation; PVI: pulmonary vein isolation; NR: not reported; ECG: electrocardiogram; global S-LAs: average mean values for peak strain during ventricular systole (S-LAs) obtained from the 4- and 2-chamber views; ePCWP: estimated pulmonary capillary wedge pressure; STE: speckle tracking echocardiography.

* The analysis included only the structured normal heart patient population.

† Only the 160 patients included for outcome analysis are depicted in this table.

### Data extraction

Data extraction was performed using a standard form by two investigators (ETOC, OMPS) and cross-verified by a third (ETM). Extracted data included: 1) First author's last name, publication year; 2) Characteristics of included studies: number of patients, region of the study, study design, ablation strategy, method of LA stiffness measurement, method of AF detection, length of follow-up period, length of blanking period and main findings; 4) Outcome results: HR and 95% CI of LA stiffness as a predictor of AF recurrence in multivariate analysis.

### Statistical analysis

The association between AF recurrence and LA stiffness following RFCA was measured by HR with 95% CI. Adjusted HRs were used, since all the studies included in the quantitative analysis employed multivariate analysis by Cox proportional hazard model to adjust for potential confounders. Log of the HR was obtained by calculating their natural logarithms. Then, standard errors were determined from the logarithmic scale and corresponding 95% CIs. The inverse variance method was used to weigh studies for the combined overall statistics. Statistical significance was defined at p-values < 0.05. Heterogeneity between studies was assessed using the Cochran's Q test and I² statistics and then evaluated by I² values. I² values less than 30% were defined as low heterogeneity; less than 60% were considered moderate heterogeneity; and above 60% defined as high heterogeneity.^8^ The random-effects model was chosen because of the different methods of LA stiffness measurements, what could lead to heterogeneity. Sensitivity analysis was done by leaving out studies and checking the consistency of the overall effect estimate. A meta-regression was not done because of the small number of studies included. The results are presented in a forest plot with 95% CI. Publication bias was verified by a funnel plot, although only 3 studies were included, which made the interpretation more difficult. All analyses were done using Review Manager 5.3 software.

## Results

### Study selection

**Figure 1:**
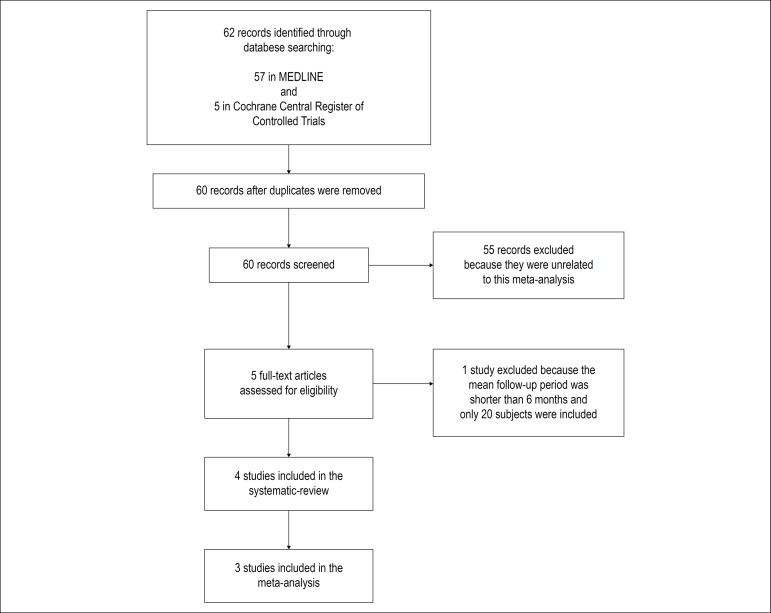
Flow diagram of the study selection.

Initially, a total of 62 studies were identified in the databases, 57 in PubMed and 5 in the Cochrane Central Register of Controlled Trials. In the duplicate analysis, we identified 2 duplicates, which were then excluded. After a careful reading of the titles and abstracts, 57 of 62 studies were excluded because they were not related to the present review. The full texts of the five studies were analysed, and 4 of them included in the qualitative analysis. The study excluded, by Marino et al.^9^ analysed only 20 patients and the mean follow-up period was shorter than 6 months. For the quantitative analysis, one full-text article was excluded because it did not report HR and 95% CI of LA stiffness as predictors of AF recurrence.^10^ Finally, four studies were included in the qualitative analysis and three in the quantitative analysis. The flow diagram of the study selection is depicted in Figure 1.

### Characteristics of the included studies

Four studies were included in this review,^10-13^ all of them prospective single centre case series studies (Table 1). The study of Machino-Ohtsuka et al.^11^ included 155 patients, and in the study by Khurram et al.,^13^ 160 patients from the original study were included in the analysis of the outcomes, and hence included in the present review. The study of Park et al.^12^ analysed 1,038 patients, however only 334 patients had a structurally normal heart and were included in the analyses. Although Kawasaki et al.^10^ analysed 137 subjects, only 109 patients underwent first ablation, and were included in the present review. Overall, 758 and 649 patients were included in our qualitative and quantitative analysis, respectively. The mean follow-up period ranged from 10.4 to 33.8 months. Studies used different techniques to measure LA stiffness, which are depicted in Table 1. All studies performed pulmonary vein isolation as ablation strategy and Holter monitoring for diagnosing AF. Also, three^10-12^ of four studies used electrocardiogram (ECG) to perform the diagnosis. Khurram et al.^13^ did not perform an ECG, although they also used 30-day event monitoring. Blanking period for AF recurrence post-RFCA lasted three months in two studies,^11,13^ one month in one study,^10^ and was not mentioned in the study by Park et al.^12^ Characteristics from all included studies are summarized in Table 1.

### LA stiffness as a predictor of AF recurrence

Two^11,13^ of the four included studies found that LA stiffness was the most important predictor for recurrence of AF post-ablation on a multivariate analysis, among several factors such as LA volume and persistent AF.

Khurram et al.^13^ observed that LA stiffness index was an independent predictor of AF ablation outcome (HR: 8.22; 95% CI: 3.54 to 19.11; p < 0.001). Besides that, 25% of patients (40 of 160) had AF recurrence after AF ablation during a follow-up period of 10.4 ± 7.6 months. Patients with AF recurrence had a higher LA stiffness index than those without recurrence. These findings are also confirmed by the study by Machino-Ohtsuka et al.,^11^ which also showed that the patients with recurrence (29%, 45 of 155) had a higher LA stiffness than those without recurrence during a follow-up period of 33.8 ± 12.2 months. In addition, the study also showed that a higher LA stiffness index was an independent predictor of recurrence of AF (HR 2.88; 95% CI 1.75 to 4.73, p < 0.001).

Also, Park et al.^12^ showed that in a follow-up period of 16.7 ± 11.8 months, a low LA compliance was associated with a two-fold increased risk of AF recurrence. Also, in the multivariate analysis, adjusting for several factors, LA stiffness was the second most important predictor for AF recurrence after RFCA (HR), only behind persistent AF.

Kawasaki et al.^10^ showed that in patients submitted to the first or second ablation, the recurrence group had a significant higher LA stiffness than the group with a successful ablation. However, in the multivariate analysis, when analysing patients undergoing the first RFCA, LA stiffness index was not a significant predictor of AF recurrence (OR).

### Meta-analysis

**Figure 2:**
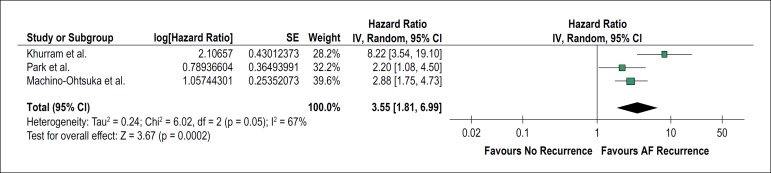
Forest plot showing left atrial stiffness as a predictor of atrial fibrillation recurrence after radiofrequency catheter ablation.

**Figure 3:**
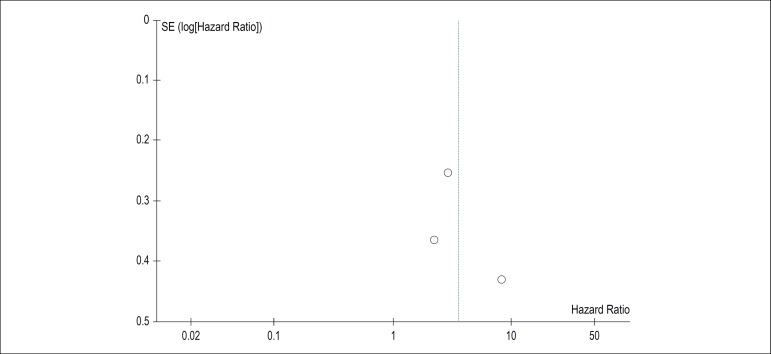
Funnel plot showing no publication.

This meta-analysis showed that LA stiffness is associated with a higher AF recurrence after RFCA (HR = 3.55, 95% CI 1.75-4.73, p = 0.0002), as shown in Figure 2. The heterogeneity test showed that there were significant differences between studies (p = 0.05, I² = 67%). The sensitivity analysis, performed to find the origin of the heterogeneity, revealed that, after removing the study by Khurram et al.,^13^ who used cardiac magnetic resonance to measure LA stiffness, there was no significant heterogeneity across the studies (p = 0.55, I² = 0%). However, the overall outcome remained the same (HR = 2.64, 95% CI 1.75-3.97, p < 0.00001). A funnel plot (Figure 3) was used to verify the existence of publication bias. There was no obvious asymmetry, suggesting that there was no publication bias.

## Discussion

As mentioned before, catheter ablation has been increasingly considered as a first-line therapy, and therefore, the importance of screening factors has also increased. This systematic review shows that in two^11,13^ of four included studies, the LA stiffness was the single most important predictor for recurrence of AF post-ablation on a multivariate analysis, among several factors such as LA volume and persistent AF. Moreover, this meta-analysis, including three studies, showed that LA stiffness is a strong predictor of AF recurrence after RFCA (HR = 3.55, 95% CI 1.75-4.73, p = 0.0002). Therefore, the use of LA stiffness in a preoperative routine may be useful for a close follow-up of patients with higher risk of developing the SLAS and AF recurrence.

### AF and stiffness of the left atrium

Previous studies have shown, despite some limitations, that patients with paroxysmal AF have increased LA stiffness.^14,15^ Also, structural remodelling caused by AF leads to LA fibrosis,^16^ which may also be a mechanism of LA stiffening. Therefore, an increase in LA stiffness could be an important mechanism of AF genesis and propagation or a consequence of AF episodes.

### Extensive Catheter Ablation

Previous studies have shown that completely circumferentially scarred pulmonary vein by RFCA was associated with less AF recurrence.^17,18^ Also, the more scarring overlaps fibrosis, decreasing the amount of unablated fibrotic tissue, the better the arrhythmia free survival.^19^ Thus, an extensive ablation appears to be the best option to reduce AF recurrence. However, in a previous study, LA scarring was associated with the development of the SLAS,^5^ leading to poor clinical outcomes post-RFCA.

### LA stiffness as a screening factor for catheter ablation

In 1988, Pilote et al.^20^ described a condition in patients undergoing mitral valve surgery for LA scarring, characterised by loss of LA compliance, pulmonary hypertension, LA dysfunction and new-onset dyspnea, the so-called SLAS.^5^ Subsequently, this syndrome was also reported by Gibson et al.^3^ in patients undergoing RFCA, with a relatively rare occurrence (1.4%). Patients with a low-compliant left atrium before the ablation may be more susceptible to develop the SLAS, as RFCA is related to an increase in LA stiffness,^21^ probably because the formation of multiple scars in the LA wall induced by the procedure.^22^ Therefore, patients with low-compliant left atrium could benefit from a measure of LA stiffness derived from a non-invasive assessment prior to AF ablation, as part of the preoperative screening process, or even routine assessment. This could help to prevent AF recurrence and the SLAS, and to promote a close follow-up of these patients.

Marino et al.,^9^ despite the study limitations, observed a linear relationship between left ventricular (LV) longitudinal strain and invasively measured LA stiffness (calculated during the ascending limb of the V-loop as the ΔLA pressure/ΔLA volume ratio). Since there is an association between the longitudinal deformation of the LA and the movement of the shared mitral annulus and the adjacent ventricle, information from LV longitudinal strain could be used to estimate LA stiffness.^9^ With this non-invasive measurement by a simple ECG, LA stiffness could be a potential new screening factor in the preoperative routine.

### Future studies

The present review shows a need for further studies to better understand the relation between LA stiffness and AF. First, an increase in the number of studies and in total sample could increase reliability of results. Also, a development of a standard non-invasive LA stiffness index would contribute for screening of patients which would not benefit from the ablation. Finally, further studies are also needed to investigate if LA stiffness is a real risk factor that could lead to AF development and propagation or if it is just a consequence of AF.

### Limitations

The present review has some limitations. First, in the quantitative analysis only three observational studies were included. Also, the I² test showed a high heterogeneity (p = 0.05, I² = 67%), although the overall outcome remained the same after excluding the study of Khurram et al.,^13^ which caused heterogeneity. This heterogeneity might be related to several factors. First, the study of Khurram et al.^13^ took place in North America, while the other two studies were performed in Asia. Second, although all methods used for the measurement of LA stiffness were different between studies, the study by Khurram et al.^13^ was the most varied among all in this sense, because it used cardiac magnetic resonance, and did not use ECG for diagnosing AF. Also, the study by Khurram et al.^13^ had the shorter mean follow-up period of all studies. In addition to these limitations, although adjusted HRs from multivariate analysis were used to reduce the effect of confounding variables, they cannot exclude them completely.

## Conclusions

The present review shows that LA stiffness is a strong predictor of AF recurrence after RFCA (HR = 3.55, 95% CI 1.75-4.73, p = 0.0002). Therefore, a standard non-invasive LA stiffness measure, could be routinely used prior to AF ablation, tracking patients with higher chances of AF recurrence and development of the SLAS.
